# Modelling Impulse Response Function of Functional Infrared Imaging for General Linear Model Analysis of Autonomic Activity

**DOI:** 10.3390/s19040849

**Published:** 2019-02-19

**Authors:** David Perpetuini, Daniela Cardone, Chiara Filippini, Antonio Maria Chiarelli, Arcangelo Merla

**Affiliations:** Department of Neuroscience and Imaging, Institute for Advanced Biomedical Technologies, University G. D’Annunzio of Chieti-Pescara, Via Luigi Polacchi 13, 66100 Chieti, Italy; david.perpetuini@unich.it (D.P.); daniela.cardone@unich.it (D.C.); chiara.filippini@unich.it (C.F.); arcangelo.merla@unich.it (A.M.)

**Keywords:** autonomic sympathetic arousal, functional infrared imaging (fIRI), thermal impulse response (TIR), general linear model (GLM), skin conductance response (SCR)

## Abstract

Functional infrared imaging (fIRI) is a validated procedure to infer autonomic arousal. Currently, fIRI signals are analysed through descriptive metrics, such as average temperature changes in a region of interest (ROI). However, the employment of mathematical models could provide a powerful tool for the accurate identification of autonomic activity and investigation of the mechanisms underlying autonomic arousal. A linear temporal statistical model such as the general linear model (GLM) is particularly suited for its simplicity and direct interpretation. In order to apply the GLM, the thermal response linearity and time-invariance of fIRI have to be demonstrated, and the thermal impulse response (TIR) needs to be characterized. In this study, the linearity and time-invariance of the thermal response to sympathetic activating stimulation were demonstrated, and the TIR for employment of the GLM was characterized. The performance of the GLM-fIRI was evaluated by comparison with the GLM applied on synchronous measurements of the skin conductance response (SCR). In fact, the GLM-SCR is a validated procedure to estimate autonomic arousal. Assuming the GLM-SCR as the gold standard approach, a GLM-fIRI sensitivity and specificity of 86.4% and 75.9% were obtained. The GLM-fIRI may allow increased performances in the evaluation of autonomic activity and a broader range of application of fIRI in both research and clinical settings for the assessment of psychophysiological and psychopathological states.

## 1. Introduction

Functional infrared imaging (fIRI) is a non-invasive methodology that is employed in biomedical applications to evaluate the skin superficial temperature of a body, providing spatial and temporal information in a contactless modality. This technique is widely employed to infer autonomic arousal through the modulation of cutaneous temperature, which is a known expression of the psychophysiological state of the subject. In particular, fIRI often exploits time-dependent variations of the skin temperature of the face to infer sympathetic and parasympathetic responses [1]. In fact, skin temperature is modulated by vasodilatation and vasoconstriction, which are themselves regulated by autonomic activity. Moreover, sweat secretion, which is induced by the sudomotor nerve [2], greatly influences skin temperature by increasing its heat conductivity. The amplitude of thermal changes is informative of the autonomic arousal only if there is a monotonic relation between the autonomic activity and the amplitude of the temperature oscillation. Evidence from the literature allows inferring that thermal skin variations produced by a stimulus are monotonic with the intensity of the stimulus; thus, it can be supposed that the above sentence is valid. For example, Yoshida et al. [2] demonstrated that participants exposed to white noise aversive stimuli at four different pressure levels (40 dB, 50 dB, 60 dB, and 90 dB) exhibited a decrease of the nose tip temperature, which was correlated with noise intensity [2]. 

Currently, the analysis of fIRI signals is performed by estimating descriptive statistical metrics, such as the average temperature (first moment of the distribution), higher-order moments, and derivatives of the signal. The metrics are evaluated differentially by estimating changes with respect to a baseline period in a specific region of interest (ROI, a group of pixels that could move in the image with subject movement). However, the employment of more sophisticated mathematical models for the analysis of fIRI signals could provide a powerful tool to detect autonomic activity and allow an in-depth investigation of the mechanisms underlying the autonomic arousal. These improvements could be effective for psychophysiological and psychopathological application. Since fIRI deals with temporal variations in space (constituted by an image with *m* × *n* pixels), mathematical models could refer to either time, space, or a combination of time and space for characterizing autonomic response.

The present study focused on modelling the temporal characteristics of fIRI in a single ROI, (e.g., centred on the nose tip). Moreover, fIRI impulse response (thermal impulse response, TIR) to sympathetic activating stimulation was characterized in order to apply a linear statistical model and to infer sympathetic activity (general linear model, GLM). Indeed, in order to employ mathematical models, it is necessary to test whether the assumptions underlying the model are satisfied. In fact, a statistical model is valid insomuch as its assumptions hold; otherwise, deduced inferences will be biased or even invalid. In order to apply GLM, the linearity of fIRI and time-invariance of TIR were tested. A linear system exhibits greater response, which is associated with a larger stimulus or activation in a linear relation. Moreover, responses are scaled versions of a template; the amplitude of each response is not affected by the previous one, and the shape and amplitude of the response do not change to the same stimulus at different administrations (i.e., linearity and time-invariance assumptions) [3,4,5,6,7]. 

The compliance of a given system with linearity and time-invariance assumptions allow the application of the GLM to such a system to represent its output. The GLM models a time series as a weighted sum of one or more known predictor variables (e.g., the onset and offset of an experimental stimulation) plus an unknown error term, in order to evaluate the extent of the contribution of the predictors to the variability observed in the time series. The unknown weights (*βs*) of each predictor are informative of the association with the original time series. For example, the GLM is extensively applied in functional neuroimaging for the evaluation of brain activity given a set of stimuli [8,9,10,11]. This method provides information about the task-related brain activation and enables the statistical evaluation of the effect of each stimuli, as well as comparisons between different experimental phases at the single-subject level. For autonomic activity evaluation and inference, the GLM is a validated approach when applied to the skin conductance response (SCR) [12].

SCR measures skin conductance, which varies with the state of the sweat glands in the skin. Since sweating is controlled by the sympathetic nervous system [13], skin conductance is an indicator of psychological or physiological arousal. If the sympathetic branch of the autonomic nervous system is highly aroused, then sweat gland activity increases, which in turn increases skin conductance. The GLM applied to the SCR (GLM-SCR) is in fact considered a gold standard procedure for autonomic activity assessment. So far, many studies have focused on the relationship between SCR and fIRI signals and their capability of investigating autonomic activity. A strong correlation between fIRI and SCR signal components was found [14,15]. However, the SCR requires contact with the skin, and, for example, it is not applicable on the hand in all dexterous tasks, since the placement of the electrodes on the fingers does not allow a comfortable execution of the task [15]. Hence, with respect to the SCR, fIRI provides some experimental advantages, including complete preservation of the ecological setting thanks to its contactless features. Nonetheless, the SCR enables the detailed quantification of autonomic responses through appropriate modelling, whereas such an approach is still lacking for fIRI.

Thus, the development of a refined method of analysis for fIRI (GLM-fIRI) may allow the diffused application of such a procedure that is at least as analogous to the SCR. Once the linearity and time-invariance of fIRI and TIR were proved, in order to establish its performance, the GLM-fIRI was compared to the GLM-SCR. 

## 2. Method

### 2.1. Participants

A total of 53 healthy participants (29 males, 24 females, mean age ± standard deviation, STD: 24.8 ± 6.3 years, range 19 to 30 years old) were recruited for the study. Participants with circulatory disease that could impact the thermal measurement were excluded. Moreover, patients exhibiting Raynaud’s phenomena and diabetic polyneuropathy were not included in the experiment, since these diseases could impact skin temperature oscillations [16]. The study was conducted in agreement with the principles described in the Declaration of Helsinki, and it was approved by the Research Ethics Board of the local university. Informed consent form was signed by all of the participants before the experiment, and they were able to withdraw from it at any time.

### 2.2. Experimental Design

**Figure 1:**
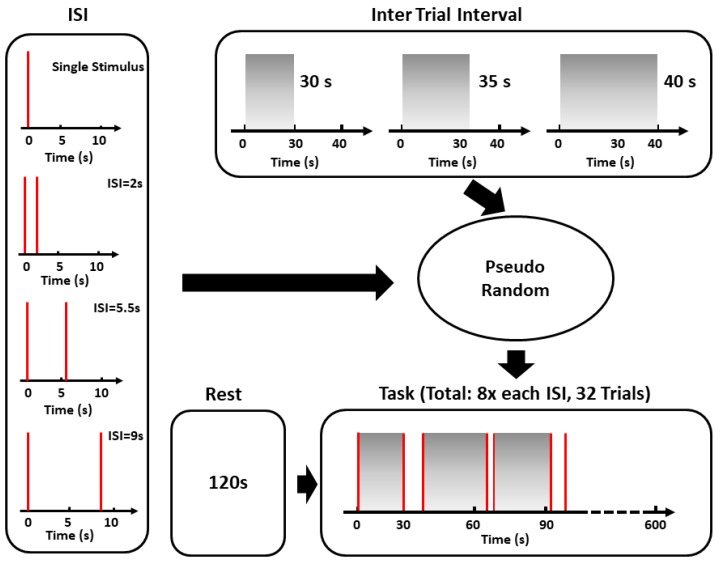
Event-related stimulation paradigm employed in the study.

An event-related stimulation paradigm was employed in accordance with previous work performed on SCR [12] (Figure 1). White noise sounds (1 s length; 10 ms onset and offset ramp; ∼85-dB sound pressure level) were delivered via a speaker in two event-related experimental conditions: single and double stimuli with an inter-stimulus interval (ISI) of 2 s, 5.5 s, or 9 s [12]. This was done to avoid subjective expectations about subsequent stimuli. The last stimulus of each trial was followed by 30 s, 35 s, or 40 s of silence. The first trial was preceded by 10 s of silence. Eight trials were realized for the single and double (eight for each ISI) stimuli conditions, for a total of 32 trials. Before the start of the white noise administration, a period of two minutes of rest was recorded.

### 2.3. fIRI Recordings and Pre-Processing

The facial temperature was recorded by means of a digital thermal infrared camera FLIR SC660 (FLIR, Wilsonville, OR, USA) (640 × 480 bolometer FPA, sensitivity/noise equivalent temperature difference: <30 mK @ 30 °C, field of view: 24° × 18°). The camera was placed 60 cm from the participant, and pointed toward the face of the subject. The sample frequency was 10 Hz. To remove the effects related to the potential drift/shift of the sensor’s response and optical artifacts, the camera was blackbody-calibrated. Standard guidelines for thermal measurements were followed during the acquisition. These guidelines recommend performing fIRI measurements in a thermoneutral environment to avoid thermoregulatory-induced alterations. Moreover, a period of acclimation for the patients is requested before the actual experiment (15 min in this study) in order to reach a condition of thermal equilibrium of the body with the environment [17,18].

**Figure 2:**
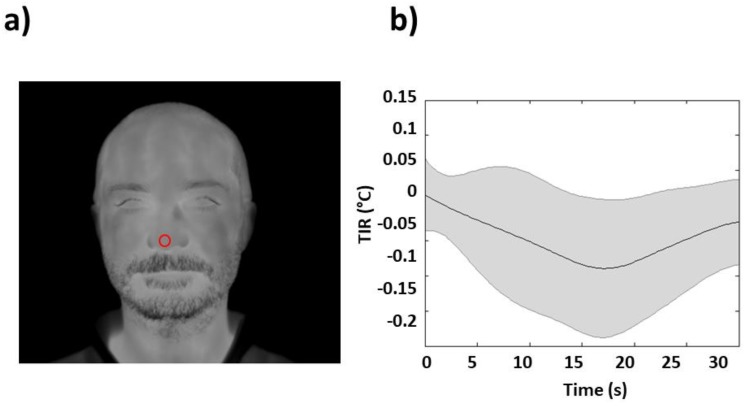
(**a**) Example of selected region of interest (ROI) (red circle) on the nose tip of a particular subject overlaid onto the subject first frame of functional infrared imaging (fIRI) recording. The ROI moved together with the relative nose tip movement within each fIRI sample image employing a soft-tissue tracking algorithm. Average temperature within the ROI was extracted as a function of time for further analysis. (**b**) Grand average, and associated STD, of fIRI response to a single white noise stimulus.

The quality of recorded fIRI was checked by visual inspection. No video was rejected. One ROI was selected, which was centered on the nose tip of each subject (Figure 2a). This ROI moved together with the relative nose tip movement within each fIRI sample image employing a soft-tissue tracking algorithm [19]. 

When the tracking algorithm failed (e.g., because of too much head rotation), the failure was displayed as a large variance of the extracted signal, which was corrected by visual inspection by substituting contaminated samples with the mean value of six samples before and after the period. The average artifact-corrected temperature within the selected ROI was considered indicative of autonomic activity [1].

The fIRI signals were filtered with a zero-lag third-order Butterworth low-pass filter (0.4 Hz) to eliminate the high-frequency oscillations that were unrelated to autonomic modulations [20]. Each subject’s time series was then z-transformed (subtracted by their average value and divided by their STD) [21] to account for between-subjects variance in fIRI amplitude. Each TIR was extracted based on a period of 30 s following each event, and was employed for further analysis as indicative of the stimulus-evoked response (Figure 1) [2]. The average single stimulus task-induced fIRI (single stimulus TIR) is reported in Figure 2b.

### 2.4. SCR Recordings and Pre-Processing

**Figure 3:**
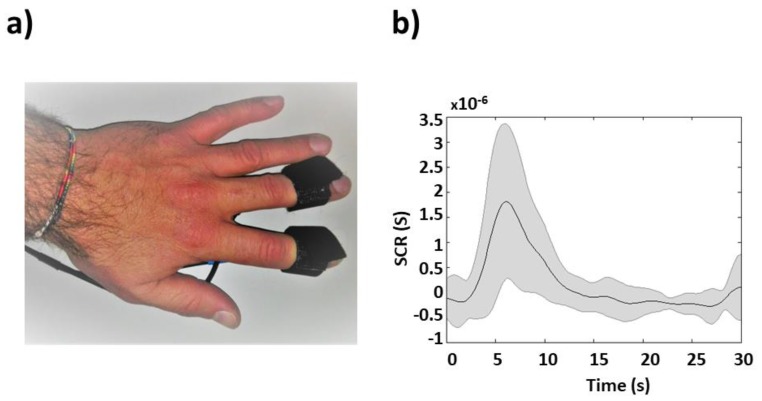
(**a**) Placement of finger electrodes for skin conductance response (SCR) measurements on the thenar/hypothenar muscles of the non-dominant hand. (**b**) Grand average, and associated STD, SCR to a single white noise stimulus.

Skin conductance was recorded on the thenar/hypothenar muscles of the non-dominant hand [22] using the AD Instrument Powerlab system, which provided a galvanic skin response (GSR) amplifier with low voltage, 75-Hz AC excitation, and automatic zeroing. The finger electrodes were made by brightly polished stainless steel, and were held with Velcro tape (Figure 3a). The sample frequency was 1 kHz. 

The SCR signal was filtered with a zero-lag third-order Butterworth bandpass filter (0.01–5 Hz) [12] and then down-sampled to 10 Hz to be homogenized with the fIRI. The tonic and phasic components of the signal were separated using a continuous decomposition analysis provided by Ledalab, which is a Matlab-based software [23]. The phasic component was then z-transformed. The average task-induced SCR is reported in Figure 3b.

### 2.5. Linearity and Time-Invariance Testing

A principal component analysis (PCA) was performed in Matlab for each subject among different single stimulus TIRs to provide a response function that explained the maximum single stimulus inter-trial variance (first principal component, first PC). 

The fIRI signal was assumed to be the output of a linear system [24]. This implies that TIRs were scaled versions of a template, and that the fIRI signal in response to different stimuli (system inputs) is given by the sum of each TIR. The event-related stimulation paradigm that was employed was suited to test these assumptions [12,25]. By evaluating the effect of ISIs on the fIRI response, it is possible to investigate the system linearity. For example, the nonlinear features of the system imply that the TIR to the second stimulus depends on the response to the first, if they occur sufficiently close to each other. 

In order to investigate the possible influences of ISIs on the TIR, double events at different ISIs were employed. The first PC was convolved with two one-stick functions, where sticks corresponded to stimulation events, and GLM was employed using two predictors (representative of each stimulus) to estimate βs relating each stimulus to the measured fIRI. Two-way repeated measures analysis of variance (rANOVA) was performed on transformed βs, considering ISI (ISI = 2 s, 5.5 s, 9 s,) and repetition (first and second stimulation) as factors (3 × 2 rANOVA). In fact, before performing the rANOVA, the normality of the βs was checked. Since they were not normally distributed (they were strictly positive), a logarithm transformation was applied. The Shapiro-Wilk test showed that the transformed data were normally distributed for each of the groups that were considered (for all of the six 3 × 2, groups, *p*s N.S.).

The statistical analysis was run in SPSS 24.0 (SPSS Inc., Chicago, IL, USA). 

The TIR was also assumed to be time-invariant. In order to test the time-invariance of the system, between trials, the TIR variance that was explained by the first PC was evaluated on event-related responses to single stimuli. In the assumption of time invariance, the TIR residual variance (variance not explained by the representative response) should be statistically smaller than the fIRI variance measured without a stimulation, i.e., at rest. 

The fulfilment of these predicted features guarantees the applicability of a GLM approach. In fact, it enables the generalization of a stereotyped TIR across individuals, thus allowing the employment of a canonical response function (CRF) for TIR, which is then convolved with the stimulus to build a predictor. 

### 2.6. TIR Modelling and CRF

After evaluation of fIRI linearity and the time-invariance of the TIR, a CRF was developed. It was analytically modelled as an exponentially modified Gaussian function in similarity with the procedure employed for modelling SCR [12]. In fact, since it is known that the mechanisms that modify skin conductance are also responsible for temperature changes, it is plausible to assume that their impulse response function could be similar in shape, although with different parameter values. 

The CRF was modelled as a Gaussian smoothed bi-exponential function (Equation (1)).

(1) $${\tilde{h}}_{can}\left(t\right)=-\left[N\left(t\right)⨂\left(E_{1}\left(t\right)+E_{2}\left(t\right)\right)\right]=-\int_{-\infty }^{+\infty }N\left(t-\tau \right)\left(E_{1}\left(\tau \right)+E_{2}\left(\tau \right)\right)d\tau$$

where ⨂ is the linear convolutional operator, *N(τ)* is a Gaussian function of standard deviation *σ* centred at *τ* (Equation (2)), and *E(t)* represents the exponential functions (Equation (3)).

(2) $$N\left(t\right)=\frac{1}{\sqrt{2\pi \sigma }}e^{-{\left(t-\tau \right)}^{2}/2\sigma^{2}}$$

(3) $$E_{1}\left(t\right)=e^{-\lambda_{1}t};\;E_{2}\left(t\right)=e^{-\lambda_{2}t}$$

The CRF was then fitted to the first PC that was extracted from single-stimuli TIRs. For the optimization of CRF parameters, a least-square iterative minimization approach was performed in Matlab environment.

### 2.7. Comparison of GLM-fIRI and GLM-SCR

Using matrix notation, the GLM is expressed as:

(4) Y=Xβ+ε

where *Y* is a *n* × 1 column vector representing the investigated time-series; *X* is a *n* × *p* design matrix, with each column representing a predictor variable of length *n*; β is the *p* × 1 column vector of the unknown weights of each predictor that indicate the strength of the association with *Y*; and *ε* is an *n* × 1 column vector that represents the residual error and is treated as a random variable. The estimation of the unknown parameter β, which is performed based on a least-square regression approach, gives information regarding the amount of variance of the signal explained by the predictor, and also enables statistical inference [8].

In order to assess the autonomic activity identification performance of statistical inference based on the GLM-fIRI, its results were compared with the GLM-SCR, assuming the GLM-SCR as the gold standard procedure. The GLM-SCR was employed in accordance with a validated CRF defined by Bach et al. [12]. Each fIRI and SCR response in a 30 s window (composed of single or double stimulus) was fitted with a predictor either based on the convolution of fIRI-CRF or on the convolution of SCR-CRF with the stimulus events. A t-statistic was estimated within each stimulus based on the computed *β* and associated *ε*, after accounting for the appropriate degrees of freedom. The null hypothesis was rejected (i.e., *β* was statistically different from zero, significant autonomic activation) with a type-I error probability of 5% (significance level at *p* < 0.05).

## 3. Results

### 3.1. Linearity 

Principal component analysis was performed on each subject employing single-stimulus event-related TIRs (eight TIRs for each of the 53 participants) (average response displayed in Figure 2b). Then, the first PC was convolved with double-events functions, and the *β* values were computed for each subject for the double-event stimuli, as described in the methods section. A total of 1272 pairs of *βs* for first and second TIRs (eight trials × three ISIs × 53 subjects) were obtained. Further statistical analysis was performed only on *βs* where a statistically significant activation was found. A total of 303 pairs of *βs* were fed to the rANOVA. Based on the rANOVA, no significant main effect or interaction of the factors considered were obtained. In particular, neither a main effect regarding ISI (F (2,200) = 2.419; N.S.) nor an interaction regarding ISI and repetition (F (2,200) = 1.933; N.S.) were found.

### 3.2. Time-Invariance 

Principal component analysis was performed on all the subjects’ TIRs concatenating the single-stimulus event-related TIRs of each subject (424 TIRs, eight TIRs for each of the 53 participants). Principal component analysis revealed that the first PC explained 61.7% of the total variance, with a residual variance of 38.3%. This residual variance was compared with the percentage of variance that was evaluated during the rest period with respect to the task period, which was 44%. The higher variance at rest with respect to the residual variance during the task were compared, employing a F-statistic where the degrees of freedom of the denominator (which should be the number of samples of signal-1, i.e., (30 s × 10 Hz)-1, 300 samples-1, 299) were corrected for the average autocorrelation of the signal to account for the assumption of the statistical independence of samples. The average full-width half-maximum of the spectrum of the signal was estimated at 12 samples, thus obtaining an equivalent number of samples of 300/12 = 25 and degrees of freedom of df = 24. A significant higher relative variance during rest was obtained (F(1,24) = 3.79, *p* < 0.05) with respect to the residual variance of the PCA analysis.

### 3.3. TIR Modelling and CRF

The CRF was modelled as a Gaussian smoothed bi-exponential function. The parameters of the analytical form of the CRF were estimated by fitting the model to the first PC that was computed during task periods. The fitting procedure was conducted employing a bi-square robust regression [26]. The estimated parameters are reported in Table 1.

**Figure 4:**
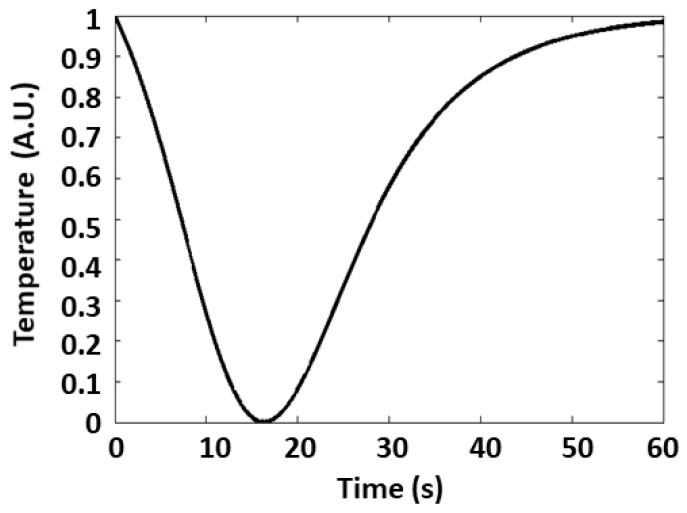
CRF of TIRs evaluated on the first principal component (PC) of fIRI in a 30 s window after a single aversive stimulus.

Importantly, these CRF parameters are known to have an approximate biophysical meaning: *τ* defines the event-to-peak time, *σ* relates to the rise time, and the two exponential constants approximately define the temporal decay. Figure 4 shows the obtained CRF shape normalized between zero and one.

### 3.4. Comparison of GLM-fIRI and GLM-SCR

**Figure 5:**
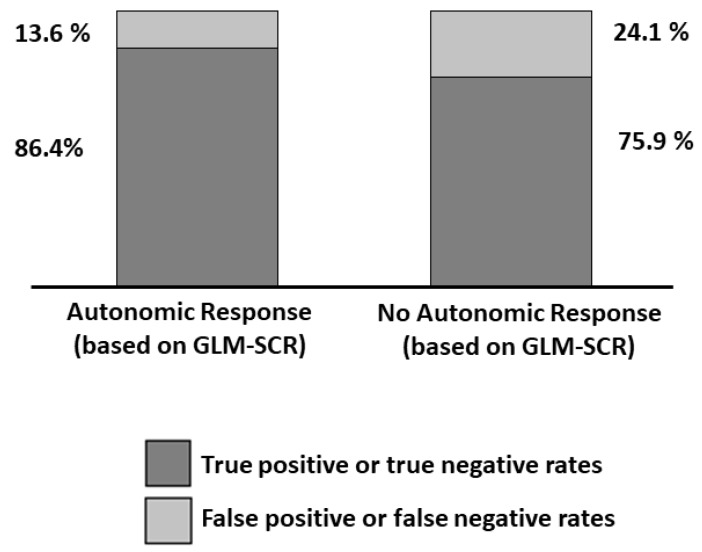
Classification outcome of GLM-fIRI when assuming GLM-SCR as the ground truth for stimulus-induced autonomic activation identification.

As described in Section 2.7, the GLM-fIRI results were compared to statistically significant activations of the GLM-SCR, assuming the GLM-SCR as the gold standard procedure. This meant that only the events detected by the GLM-SCR technique were considered indicative of autonomic arousal. The results of this analysis are described through the confusion matrix reported in Table 2 and Figure 5. 

## 4. Discussion

In this paper, the feasibility of a GLM-based analysis to fIRI thermal signals (GLM-fIRI) for the identification of autonomic response was tested. As a first step, the two assumptions of linearity and time-invariance for the employment of GLM were tested on fIRI and its associated TIR to sympathetic activating stimuli. The results suggested that the shape and amplitude of the response was indeed not dependent on the ISI. Particularly, the response to double stimuli close in time could be modelled as the sum of two single responses. This characteristic is typical of linear systems, thus proving fIRI linearity, at least in first approximation. Moreover, by applying a PCA on single-stimulus TIRs, the first PC could explain 61.7% of the total variance, in which the associated residual variance of 38.3% was found to be statistically lower than the variance of fIRI that was associated to spontaneous physiological fluctuations at rest. This result suggested that a single response model (first PC) was indeed able to explain a large amount of each single TIR’s variance, thus proving the time-invariance characteristics of TIRs for practical purposes. After proving the linearity and time-invariance of fIRI, a CRF was modelled for the employment of GLM through an iterative least-square approach.

With respect to CRF characteristics, an optimized set of parameters was found to provide a negative TIR. This is compatible with the decrease in temperature caused by the sudomotor activity. However, it should be highlighted that the developed GLM procedure may indeed allow an opposite response (positive TIR), which would provide a negative *β*. Importantly, the identified parameters of CRF are compatible with the temporal dynamics of fIRI [1].

The statistical inference of fIRI based on GLM (GLM-fIRI) was compared to the gold-standard inference of autonomic arousal based on the GLM that was applied to the SCR (GLM-SCR). By assuming the GLM-SCR the ground truth, the GLM-fIRI provided a sensitivity of 86.4% and a specificity of 75.9% to the autonomic arousal. However, a 100% sensitivity and specificity of SCR is indeed an approximate assumption, and GLM-fIRI sensitivity and specificity should require further assessment.

Importantly, GLM-based analysis revealed that for some stimuli, no induced response was detected through either fIRI or SCR. This could indeed be related to an effect of response habituation, which implies system memory and peripheral and central [3] adaptation to the stimulus. However, it is worth stressing that this effect is compatible with a linear system. 

This study could pave the way for an investigation of mathematical modelling in fIRI, overcoming the current limitations of fIRI signal analysis, which is mainly based on descriptive metrics. In fact, this method could provide statistical inferences about sympathetic arousal at the single-subject level and at a higher accuracy when compared to standard approaches, whose accuracy might be useful in research and clinical settings. For example, in outpatient environments, participants’ emotional and anxiety states could impair the performance of cognitive tasks that can be performed for the assessment of cognitive pathological impairment [27,28,29,30,31]. In this context, it might be relevant to evaluate the autonomic activity in each subject and stimulation during test execution through a GLM analysis. 

It should be highlighted that the TIR to one particular aversive stimulus was investigated in the presented work; thus, further studies should be performed to characterize the TIR to other stimuli and assess a single CRF generalization and its possible application on cognitive tests and aversive stimuli presented in ecological settings. In fact, with respect to the SCR, fIRI is completely contactless; hence, it is able to preserve the ecological features of the monitoring, representing a great advantage for application in research environments as well as in clinical practice. 

Finally, to further increase GLM-fIRI sensitivity and specificity, it could be highly relevant to develop procedures to uncouple functional evoked fIRI fluctuations from fIRI changes related to spontaneous non-specific physiological oscillations.

## 5. Conclusions

In this study, the feasibility of employing the GLM on fIRI was tested in order to identify sympathetic stimulus-induced activity. After evaluation of the linearity and time-invariance of the system of interest, a CRF derived from TIRs was modelled to an aversive stimulus administered in an event-related paradigm. The experimental characterization of the GLM-fIRI was compared to the gold-standard procedure of GLM-SCR and provided good specificity and sensitivity to autonomic activation. This novel approach of characterizing a linear impulse response of fIRI and applying it in a GLM approach for studying sympathetic activity allows for a more in-depth characterization of fIRI response in physiology and a high sensitivity and specificity of fIRI in a clinical environment for the assessment of impaired autonomic modulations. These applications can be performed in a completely ecological situation thanks to the contactless characteristic of fIRI. However, further studies are necessary to better investigate TIR to different stimuli and cognitive tasks in order to expand the research and clinical field of fIRI application.

## Figures and Tables

**Table 1:** Estimated parameters for a modelled canonical response function (CRF) of thermal impulse response (TIR).

Model Parameters	Estimated Value
$\hat{\tau }$	10.3734
$\hat{\sigma }$	6.7601
${\hat{\lambda }}_{1}$	0.0765
${\hat{\lambda }}_{2}$	0.0994

**Table 2:** Confusion matrix of the general linear model with functional infrared imaging (GLM-fIRI) assuming the general linear model with skin conductance response (GLM-SCR) as the gold standard procedure for the identification of an autonomic response.

	ID	0	1	Tot
**Counts**	**0**	771	244	1015
	**1**	150	955	1105
**%**	**0**	75.9	24.1	100.0
	**1**	13.6	86.4	100.0
